# Regulation of mitochondrial ROS production by HIC‐5: a common feature of oncogene‐induced senescence and tumor invasiveness?

**DOI:** 10.1111/febs.14746

**Published:** 2019-01-25

**Authors:** Wolfgang Doppler, Pidder Jansen‐Dürr

**Affiliations:** ^1^ Division of Medical Biochemistry Biocenter Innsbruck Medical University Austria; ^2^ Research Institute for Biomedical Aging Research University of Innsbruck Austria

## Abstract

Transformation by the ras oncogene can result in promotion of metastasis as well as induction of senescence via increased tissue remodeling, for example, by matrix metalloproteases. Increased production of mitochondrial reactive oxygen species (mtROS) via NADPH oxidase 4 (NOX4) is implicated in this process. Hydrogen peroxide‐inducible clone‐5 (HIC‐5) is postulated to sense both matrix detachment of transformed cells and intracellular ROS and can inhibit ras signaling via inhibition of NOX4.

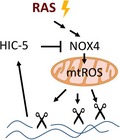

**Abbreviations**
HIC‐5: hydrogen peroxide‐inducible clone‐5
NOX4: NADPH oxidase 4
ROS: reactive oxygen species
TGFβ: transforming growth factor β

Hydrogen peroxide‐inducible clone‐5 (HIC‐5) was identified when searching for transforming growth factor β (TGFβ) and hydrogen peroxide‐inducible genes by Shibanuma *et al*. 1 from the Showa University School of Pharmacy in Tokyo. HIC‐5 contains four LIM domains and is most closely related to the focal adhesion protein paxillin. In contrast to the embryonic lethal phenotype of paxillin knockout mice, HIC‐5 null mice exhibit no obvious phenotype, at least under standard rearing conditions 2. Besides its poorly characterized function at focal adhesions, it can act in the nucleus as an adaptor to regulate transcription by binding to transcription factors involved in the regulation of cellular differentiation and cell cycle, such as SP‐1, the glucocorticoid receptor, the androgen receptor, and peroxisome proliferator‐activated receptor. Its function in modulating transcription has been particularly well studied for the glucocorticoid receptor on a genome‐wide basis 3. Intriguingly, HIC‐5 contains a nuclear export sequence (NES) with two cysteines that are susceptible to oxidization by reactive oxygen species (ROS), which triggers subsequent inactivation of the NES function, thereby leading to nuclear accumulation of HIC‐5 4. By this means, HIC‐5 can be considered as a sensor for ROS as well as a mediator of the cellular response to ROS via its nuclear function.

As now described in the paper by Mori *et al*. 5 published in this issue of the FEBS Journal, one of the actions of HIC‐5 is to downregulate NADPH oxidase 4 (NOX4), a membrane‐bound protein reported to localize to various subcellular sites, including the plasma membrane, the endoplasmic reticulum membrane, and the mitochondria. As cellular localization studies with NOX4 are usually performed by immunofluorescence assays, the choice of the appropriate antibodies is crucial, and only a few reliable antibodies are currently available 6. Downregulation of NOX4 by HIC‐5 resulted in a decrease of mitochondrial ROS levels (mtROS), in accordance with the described function of NOX4 to increase mtROS. Thus, HIC‐5 can be considered as a negative feedback regulator of mtROS in order to prevent cellular damage through excessive ROS production. Downregulation of NOX4 by HIC‐5 was already described in a previous report 7. In this manuscript, evidence was presented for a posttranslational mechanism involving ubiquitin‐proteasomal system‐mediated degradation of NOX4 promoted by the association of the HIC‐5 protein with the ubiquitin ligase Cbl‐c and the ubiquitin‐binding protein heat shock protein 27. Destabilization of NOX4 mRNA by HIC‐5 is proposed as another mode of interaction of these two proteins 5. A model explaining how NOX4 may increase mtROS production was proposed based on the finding that NOX4 inactivates complex I of the mitochondrial electron transport chain 8 in human endothelial cells; however, if this mechanism is also effective in the cell types used in the study by Mori *et al*. remains to be shown.

In the breast cancer cell line MDA‐MB‐231, the decrease of mtROS induced by HIC‐5 resulted in destabilization of the gelatin matrix metalloprotease MMP9. When HIC‐5 was knocked down in these cells, the capability of these cells to form lung metastasis was increased. This was demonstrated in NOD/SCID mice with cells orthotopically injected into the mammary gland. Consistently with the *in vivo* results, the knocked down cells exhibited increased invasive properties in *in vitro* experiments. The increased propensity to form metastasis was attributed to higher expression levels of MMP9 after downregulation of HIC‐5; of note, the growth properties of the tumor cell line were not affected by knockdown of HIC‐5.

By examination of six tumor cell lines transformed by different oncogenes, Mori *et al*. observed the inhibitory function of HIC‐5 on MMP9 expression in MDA‐MB‐231 breast cancer cells and the colon cancer cell line EJ‐1 5. Since only these two cancer cell lines, but none of the others, contain activated H‐RAS or K‐RAS oncogenes, the authors speculate that HIC‐5 requires activated RAS to mediate its effect on NOX4, a notion which was further supported by the acquisition of this phenotype by mammary epithelial cells after transformation with H‐RAS. In this respect, it may be worth to note that NOX4 gene expression is increased in RAS‐induced cellular senescence and its function is required to enforce DNA damage and subsequent RAS‐induced senescence 9, a well‐known tumor suppressor mechanism. Moreover, NOX4‐dependent senescence, induced either by an activated RAS oncogene or naturally occurring in human endothelial cells 10, involves increased secretion of a bunch of extracellular proteins (referred to as the senescence‐associated secretory phenotype ‘SASP’), including pro‐inflammatory cytokines and chemokines, matrix metalloproteases, and others. Of note, composition of the SASP depends both on the cell type involved and the stimulus used to induce senescence; although MMP‐1, ‐3, ‐10, ‐12, ‐13, and ‐14 have been described as SASP components in various cellular models, MMP‐9 was so far not identified as member of the SASP 11. Moreover, the HIC‐5 dependent signaling cascade uncovered in the current article has similarity to RAS‐induced senescence, featuring TGFβ‐Nox4 signaling, oxidative stress, and DNA damages response as shared features of both replicative and oncogene‐induced senescence (Fig. 1) 12. Future work will tell if the similarity in pathways underlying HIC‐5‐mediated suppression of metastatic behavior of cancer cells and oncogene‐induced senescence will extend beyond the currently depicted mechanisms. Other than the situation in tumor epithelial cell expressing Ras, expression of HIC‐5 in carcinoma‐associated fibroblasts has been described to promote metastasis by its positive influence on the generation of tumor‐promoting stroma 13.

The effect of HIC‐5 on metastasis and invasion appears to be dependent on the stage of tumor dissemination as suggested by the opposing results on metastasis formation reported by another group using a different experimental protocol to study metastasis. The group employed siRNA for short‐term knockdown of HIC‐5 in MDA‐MB‐231 cells and injection of cells via the tail vein. There, HIC‐5 was found to promote lung metastasis 14 rather than to inhibit metastasis. In order to explain this discrepancy, Mori *et al*. 5 speculate the prevalence of a short‐term metastasis‐promoting effect of HIC‐5 via its effect on RhoA signaling 14 followed by a long‐term protecting effect mediated by downregulation of MMP9 5.

The study by Mori *et al*. is another example for the importance of mtROS in facilitating tumor invasion and metastasis. One of the necessary steps preceding tumor invasion of transformed epithelial cells is the detachment from its matrix attachment sites, which is controlled by integrin receptors linked to the focal adhesion complex. Detachment has been shown to lead to an increase of intracellular ROS production 15 and is facilitated by epithelial–mesenchymal transition and loss of focal adhesions. Shibanuma and colleagues now propose that, under these conditions, HIC‐5 might be a key regulator for adapting cells to ROS‐mediated cellular stress. It senses increased levels of ROS through its oxidation sensitive NES as well as changes in the focal adhesion complex after matrix detachment. Both events lead to increased HIC‐5 levels in the nucleus and initiate changes in gene expression including mitigating the production of mtROS via NOX4.

**Figure 1:**
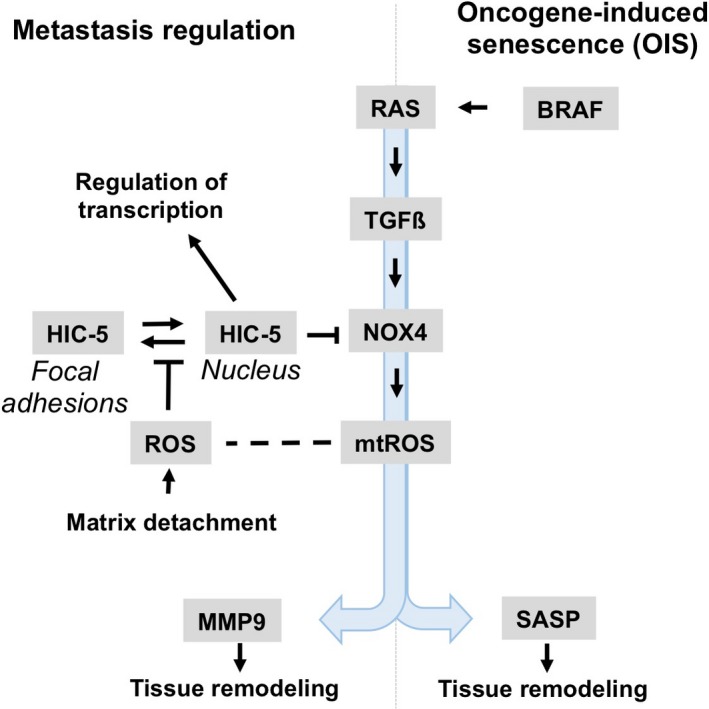
HIC‐5 in metastasis regulation and tumor invasion; potential mechanistic links to oncogene‐induced senescence. In senescence induced by the BRAF or RAS oncogenes, tissue remodeling is dependent on the induction of SASP proteins by mtROS, generated by NOX4. Mori *et al*. propose a novel pathway to regulate NOX4 expression and mtROS. It involves the adaptor protein HIC‐5 and the matrix metalloprotease MMP9 and is supposed to affect the metastatic properties of tumor epithelial cells transformed by the RAS oncogene. In their model, HIC‐5 acts as a ROS sensor via its oxidation sensitive NES. Generation of ROS is triggered after detachment of cells from the extracellular matrix (matrix detachment), a required step in metastasis. The increase of nuclear HIC‐5 results in activation of its pleotropic function as an adaptor protein to regulate transcription. The role if any of HIC‐5 in oncogene‐induced senescence remains elusive.
